# Modified mosquito landing boxes dispensing transfluthrin provide effective protection against *Anopheles arabiensis* mosquitoes under simulated outdoor conditions in a semi-field system

**DOI:** 10.1186/s12936-015-0762-8

**Published:** 2015-06-24

**Authors:** Marta Andrés, Lena M Lorenz, Edgar Mbeleya, Sarah J Moore

**Affiliations:** Department of Cellular Neurobiology, University of Göttingen, Julia-Lermontowa-Weg 3, 37077 Göttingen, Germany; London School of Hygiene and Tropical Medicine, Keppel Street, London, WC1E 7HT UK; Environmental Health and Ecological Sciences Thematic Group, Ifakara Health Institute, Bagamoyo Research and Training Centre, Bagamoyo, Tanzania; Swiss Tropical and Public Health Institute, Socinstr. 57, 4051 Basel, Switzerland; University of Basel, Petersplatz 1, 4003 Basel, Switzerland

**Keywords:** Repellent, *Anopheles arabiensis*, Semi-field system, Transfluthrin, Mosquito landing box, Outdoor malaria control

## Abstract

**Background:**

Efforts to control malaria vectors have primarily focused on scaling-up of long-lasting insecticidal nets (LLINs) and indoor residual spraying. Although highly efficient against indoor-biting and indoor-resting vectors, these interventions have lower impact on outdoor-biting mosquitoes. Innovative vector control tools are required to prevent outdoor human–mosquito contacts. In this work, the potential of spatial repellents, delivered in an active system that requires minimal user compliance, to provide personal protection against exophagic mosquitoes active in the early evening was explored.

**Methods:**

A device previously used as an odour-baited lure and kill apparatus, the mosquito landing box (MLB), was modified to dispense the volatile synthetic pyrethroid, transfluthrin, as a spatial repellent. The MLB has an active odour-dispensing mechanism that uses a solar-powered fan and switches on at dusk to provide long duration dispensing of volatile compounds without the need for the user to remember to employ it. Two MLBs were located 5 m from a human volunteer to investigate the repellent effects of a transfluthrin ‘bubble’ created between the MLBs. Transfluthrin was emanated from polyester strips, hanging inside the MLB odour-dispensing unit. A fully randomized cross-over design was performed in a large, semi-field, screened cage to assess the effect of the repellent against laboratory-reared *Anopheles arabiensis* mosquitoes under ambient outdoor conditions. The knock-down capacity of the transfluthrin-treated strips was also evaluated at different time points up to 3 weeks after being impregnated to measure duration of efficacy.

**Results:**

The protective transfluthrin bubble provided 68.9% protection against *An. arabiensis* bites under these simulated outdoor conditions. Volatile transfluthrin caused low mortality among mosquitoes in the semi-field system. Transfluthrin-treated strips continued to knock down mosquitoes in laboratory tests, 3 weeks after impregnation, although this effect diminished with time.

**Conclusion:**

Modified MLBs can be used as efficient and long-lasting dispensers of volatile spatial repellents such as transfluthrin, thereby providing high levels of protection against outdoor-biting mosquitoes in the peri-domestic space. They have a potential role in combatting outdoor malaria transmission without interfering with effective indoor interventions such as LLINs.

## Background

Recent global malaria control efforts have substantially reduced malaria morbidity and mortality, thanks to the scaling-up of highly effective vector control strategies, mainly indoor residual spraying (IRS) and long-lasting insecticidal nets (LLINs), coupled with improved diagnosis and effective treatment with artemisinin-based combination therapy (ACT) [1]. While LLINs and IRS are outstanding vector control tools, they are insufficient to control all malaria vector mosquitoes. LLINs are highly effective against indoor (endophagic) night-biting mosquitoes, while IRS is effective against mosquitoes that rest indoors (endophilic) and both are most effective against mosquitoes that mainly feed on humans [2]. However, malaria is transmitted by multiple members of the genus *Anopheles* that vary in the time and place of biting as well as host choice [3]. Some *Anopheles* vectors exhibit outdoor-biting and resting behaviours, thereby effectively avoiding contact with LLINs and IRS. This has led to an increase in the relative abundance of outdoor-biting mosquitoes as compared to strictly indoor-biting mosquitoes in recent years, which continue to maintain a lower level of malaria transmission [4]. In Tanzania and Kenya, proportions of indoor-biting *Anopheles gambiae* and *Anopheles funestus* has decreased significantly in recent years, as a consequence of large-scale LLIN coverage, while the relative proportion of exophagic *Anopheles arabiensis* has increased [5–7]. In the Mekong and Amazon regions, a large proportion of malaria transmission occurs outdoors or in the evening before people go to bed under bed nets [4, 8] and many vectors do not rest indoors [9, 10], thus limiting the efficacy of conventional indoor mosquito control interventions.

The increasing awareness that malaria eradication is not feasible without tackling outdoor-biting vectors [11] has focused attention onto innovative approaches for residual malaria control. These include odour-baited traps [12, 13], spraying insecticides onto livestock [14], use of larvicides [15–18], environmental management [19, 20], low-cost topical repellents [21], and use of spatial repellents [22]. Spatial repellents are already one of the most commonly used household insecticidal products worldwide [23] and act in the volatile phase to decrease human–vector contact [24–26]. Mosquito coils with transfluthrin [27] and metofluthrin [28] have been shown to prevent malaria infections when used indoors in areas with early-biting mosquitoes. However, to provide personal protection outdoors, new dispensing methods should be explored to disperse spatial repellents efficiently over the peri-domestic space in outdoor conditions without need for daily user compliance [29]. User compliance has been shown to be a key determinant of the effectiveness of malaria interventions. Although topical repellents effectively protect individuals from mosquito bites [30, 31] their efficacy as a malaria prevention tool varies [32–36], presumably because of on the requirement of daily user compliance [36]. Long lasting repellent interventions that require minimal user compliance are necessary to overcome this limitation.

One such solution may be provided by the mosquito landing box (MLB), which was originally developed to lure and kill outdoor-biting mosquitoes in Tanzania [13]. MLBs consist of a wooden box and an odour-dispensing unit with a fan on top. A deflecting dish fitted on the underside of the top cover dispenses the air drawn towards it by a fan powered by a solar-rechargeable battery, so that volatiles are equally dissipated in all four directions. MLBs are designed for use in low- and middle-income countries as they are built out of economical materials and are driven by a solar panel.

The aim of this study was to investigate whether modified MLBs can also be used to disperse spatial repellents over long periods of time, while requiring relatively low user-compliance, thereby providing an alternative to mosquito coils for spatial protection against outdoor-biting mosquitoes.

## Methods

### Mosquitoes

Laboratory-reared *An. arabiensis* mosquitoes (Ifakara strain, originally sourced in 2008, from the village of Sakamaganga in southeastern Tanzania) were used for SFS experiments and *An. gambiae* sensu stricto (*s.s.*) (Ifakara, originally sourced in 1996 from the village of Njage in south eastern Tanzania). Both strains are completely susceptible to all classes of insecticides. The colony was reared in an insectary facility at Ifakara Health Institute (IHI), Bagamoyo branch, United Republic of Tanzania. Temperature and humidity in the insectary are kept between 27 ± 2°C and 70–80%, respectively, with ambient 12D:12L hour light dark cycle. Larvae are fed on Tetramin^®^ fish food and adults are given 10% glucose solution ad libitum delivered through Whatman^®^ paper, and provided with human blood between 3 and 6 days after hatching (arm feeding). For the experiment, 3–8 days old nulliparous *An. arabiensis* females that had never blood fed were used. The mosquitoes were sugar starved for 6 h before the start of each nightly experiment, and only mosquitoes that demonstrated avidity when a human hand was placed close to the cage were selected for use.

### Adaptation of the mosquito landing boxes (MLBs)

**Figure 1:**
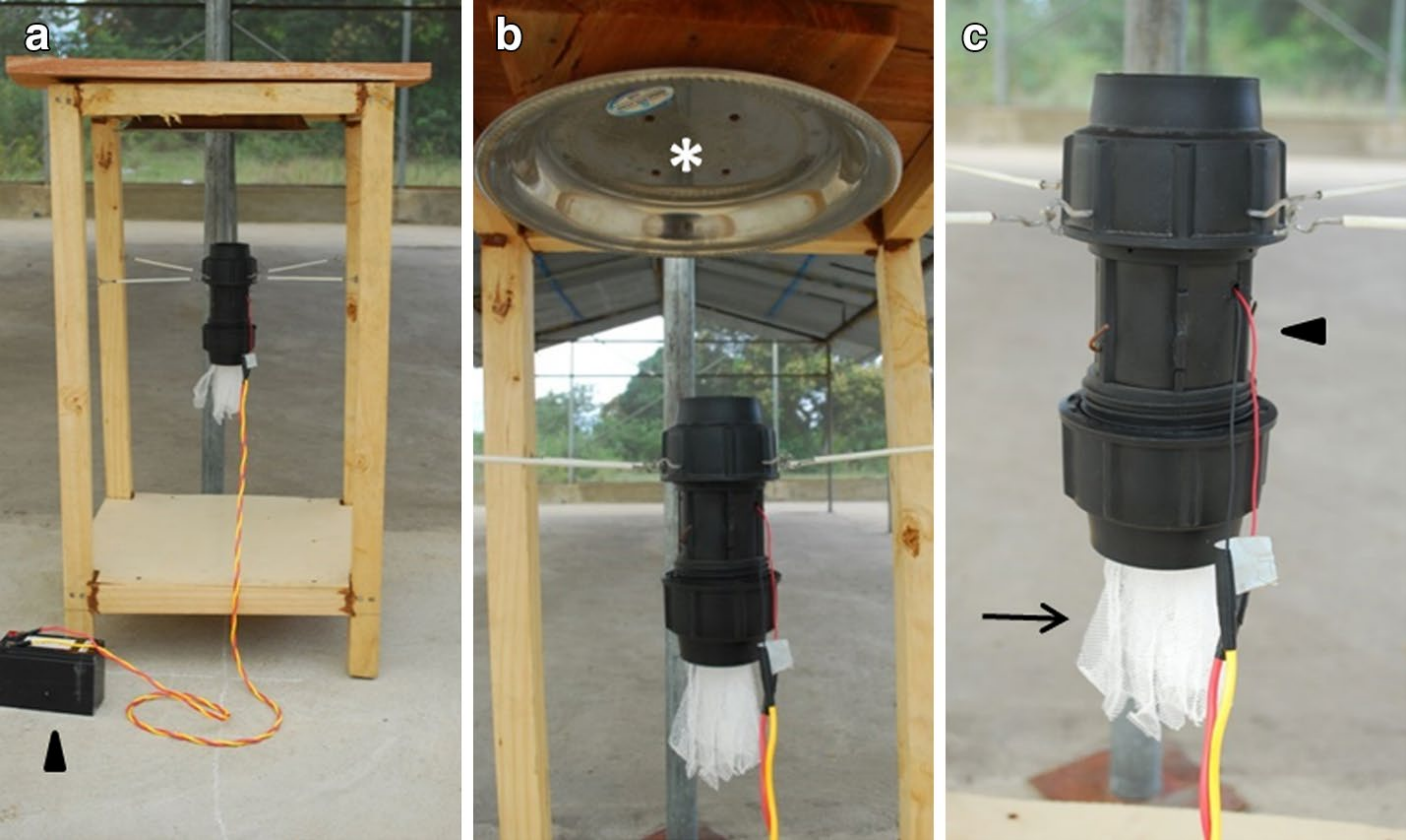
Modified mosquito landing box. **a** The lateral panels from the mosquito landing box (MLB) previously described in Matowo et al. [13] were removed to allow a better dispersion of the repellent. A solar battery (*arrowhead*) provides the energy to power the fan that draws the air emanated by the strips. **b** A deflecting dish (*asterisk*) is attached to the underside top cover to disperse the air driven by the fan. **c** The dispensing unit consists of a PVC pipe (*arrowhead*) with a fan on the top of it. The polyester strips are hanging from a cable wire within the dispensing tube (*arrow*).

The structure of the MLB has previously been described [13]. In its original design, the MLB consisted of a wooden box measuring 0.7 × 0.7 × 0.8 m on short wooden pedestals that raised it 10 cm above ground. For this study, the side panels were removed, leaving only the wooden box structure to allow for a better dispersion of the repellent volatiles (Figure 1a). The dispensing unit consists of a short PVC pipe of 5.7 cm diameter and 20 cm length held by suspension wires to the wooden frame. A 12 V fan to the top of the PVC pipe that draws air upwards through the dispensing unit (Figure 1c). The fan is powered by a 12 V, 7.2 A battery charged by a 20 W solar panel that switches on at dusk using a photoswitch. The polyester strips with repellent are hung within the PVC pipe. A deflecting dish is attached to the underside top cover of the MLB to redirect the air drawn upward by the fan in all directions (Figure 1b).

### Preparation of the polyester strips and transfluthrin solution

Polyester strips (untreated Safinet^®^, A to Z Textile Mills Ltd, Arusha, Tanzania) were used to deliver the transfluthrin. Ten strips (1 cm × 25 cm) were cut out of the untreated mosquito nets, stapled to a hooked cable wire to be hung in the PVC pipe of the MLBs. The disposition of the polyester strips maximized its surface to emanate transfluthrin.

Polyester strips were impregnated with 90 mg transfluthrin. To do so, a 1.1% transfluthrin stock solution in 70% ethanol was prepared and kept in the refrigerator at 4°C in a sealed glass bottle. Ten polyester strips were soaked every experimental day in this solution for impregnation. The strips were hung into the odour-dispensing unit of the MLBs directly after impregnation with the fans switched on to start dispersing the treatment.

### Semi-field system

**Figure 2:**
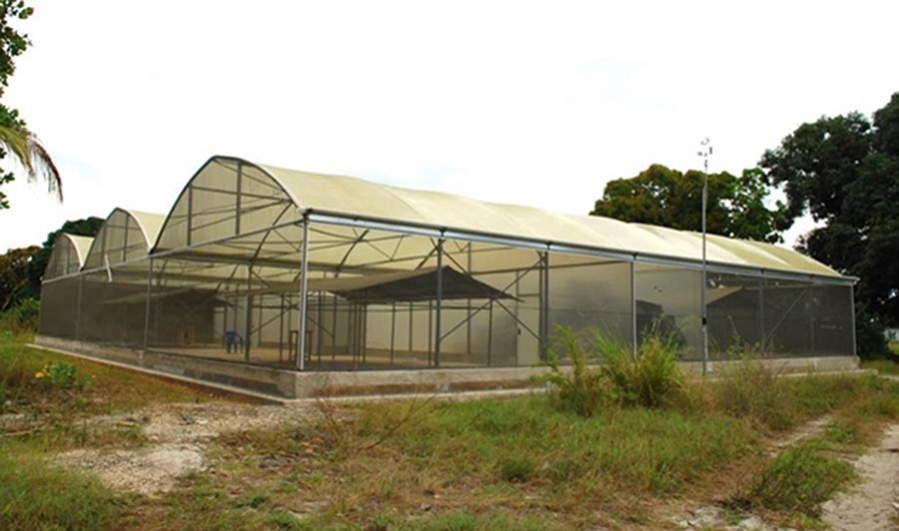
Semi-field system. The walls and the roof of the semi-field system (SFS) are made of metal frames and fiberglass netting material. The SFS has two sections separated by heavy-duty polyurethane. Each of these sections contains one experimental unit.

The study was conducted in the semi-field system (SFS) facility at IHI Bagamoyo branch, United Republic of Tanzania (Figure 2). The SFS consists of large screened cage with two sections each measuring 9 m × 28.8 m that were used for the experiment, each closed off with heavy-duty polyurethane sheeting so that each compartment is independent of the other [37]. The SFS approximates outdoor conditions of temperature, humidity and airflow [38]. Laboratory-reared mosquitoes are used in SFS facilities so experiments can be replicated within a short period of time releasing the same number of mosquitoes each night, thus avoiding bias introduced by natural fluctuations of mosquito populations. Additionally, laboratory mosquitoes are disease free which allows measurement of mosquito–human contact with no risk to human volunteers.

### Study design

**Figure 3:**
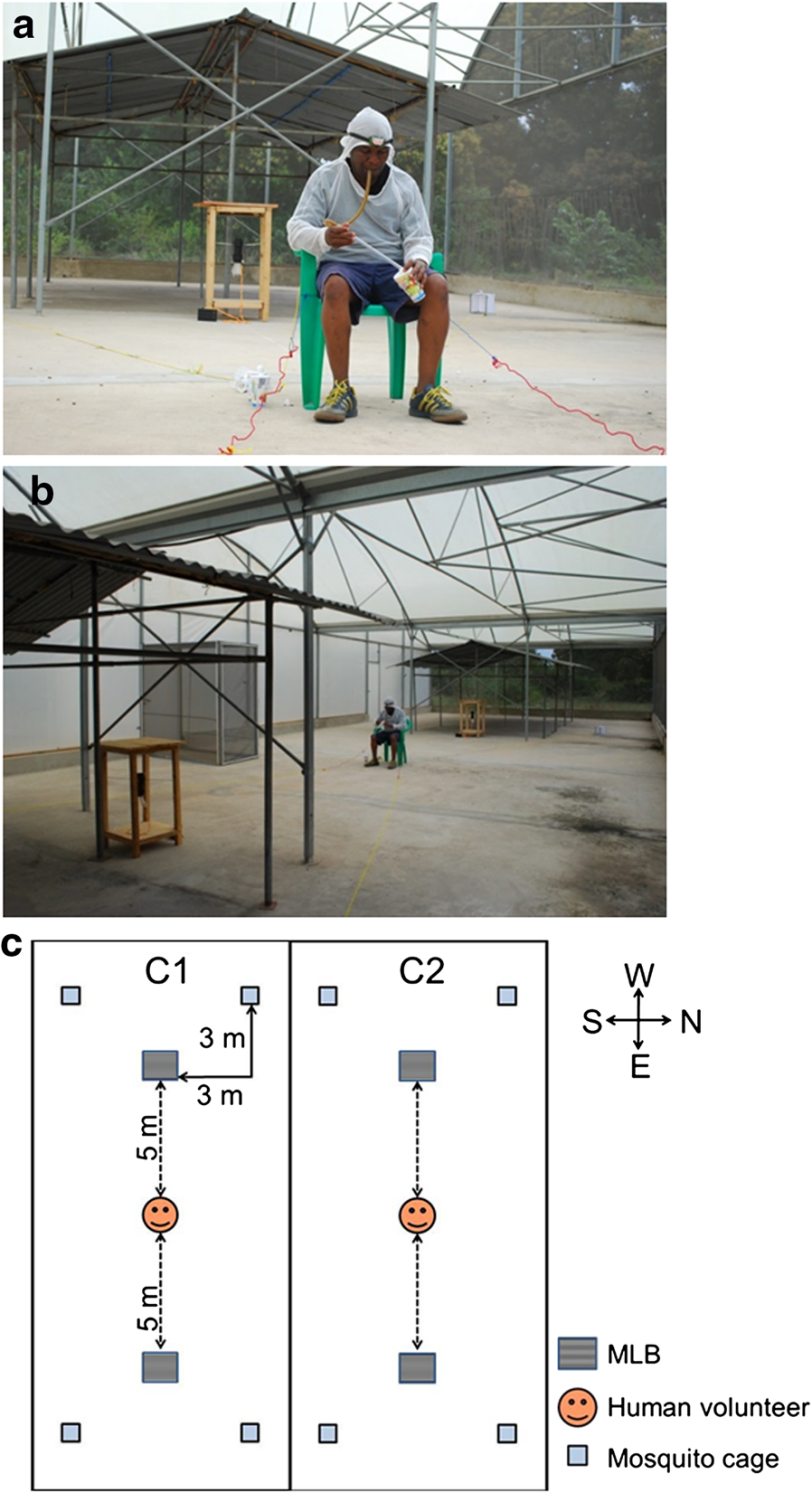
Study design. **a**, **b** Within each semi-field compartment, a human volunteer sat between the two MLBs at a distance of 5 m from each. The volunteer exposed his lower legs to the mosquito bites and was provided with a protective net to avoid mosquito bites elsewhere on the body, a torch to catch the mosquito in the darkness, a mouth aspirator to collect the mosquito and four paper cups for mosquito collection during the 4 h of the experiment. **c** Schematic of the study design. The two compartments in the SFS are located side by side. Fifteen mosquitoes were released from each of four cages (total of 60 mosquitoes) located behind the MLBs. Four plastic strings connected the chair to the cages to allow the volunteer to open the cages remotely. Each night, either treatment or control were dispersed by the two MLBs in each semi-field compartment.

A fully randomized, cross-over design was used, with either treatment (transfluthrin-impregnated polyester strips) or control (ethanol-soaked polyester strips) assigned to one of the two SFS compartments. Two MLBs were used in each SFS compartment to create a protective ‘bubble effect’ around the human volunteers [37], who sat in the middle area, 5 m equidistant from each of the MLBs, which were located 10 m apart. Fifteen mosquitoes were released from each of four cages located 3 m behind and laterally from the MLBs (60 mosquitoes, total) (Figure 3).

Four volunteers participated in the study in two rounds of experiments. Treatment or control MLBs were maintained in the same compartment for 4 consecutive days and the pair of volunteers rotated between the two compartments every night. Then the compartments were left unused for 3 days, to dissipate residual repellent. After each 3-day down-time, the treatment or control MLBs were swapped between compartments to control for positional bias. This was repeated for 4 weeks for two pairs of volunteers, resulting in 32 replicates of the experiment.

### Repellent treatments

Every experimental day at 17.25, two sets of ten polyester strips were soaked in either the treatment (1.1% transfluthrin dissolved in 70% ethanol) or control (70% ethanol) solution. Both solutions were kept in darkness to avoid inactivation of transfluthrin by light. At 17.55, after half an hour of soaking, the four sets of ten polyester strips were located in their respective MLBs within each of the compartments. Fans were turned on to allow for the dispersion of either treatment or control for 1 h before starting the experiment to create the protective bubble effect. After finishing the experiment, the four sets of polyester strips were collected and stored under ambient outdoor conditions in the shade to perform the knock-down tests described below.

### Mosquito collection

At 19.00, the volunteers released 15 *An. arabiensis* female mosquitoes from each of four cages (60 female mosquitoes in total) within each SFS compartment using strings that connected their chair to the cages (Figure 3) so that volunteers did not bias mosquito responses by moving around in the SFS. Volunteers performed human landing catches (HLCs) for 4 h until 23.00. The HLC has been the most used method to estimate human vector contact [39] and consists of human volunteers sitting with their lower legs exposed to mosquito landings and collecting mosquitoes that come to attempt to feed on them using a mouth aspirator. In the study, the volunteers wore knee-length shorts and ankle-high boots to standardize the area of the lower limbs exposed, and wore netting jackets to ensure mosquitoes could not feed on their upper bodies. All volunteers were experienced in conducting HLC and used a head torch with red light, which they switched on only when they felt a mosquito landing on their limb or when scanning the legs every 30 s for mosquitoes to minimize light affecting mosquito responses [40]. Each hour, mosquitoes were collected in a different paper cup labelled to show the time of mosquito collection. After the experiment, the number of mosquitoes in each cup was counted.

Mosquitoes were kept in the paper cups at 27°C ± 2 with access to cotton wool soaked with 10% glucose solution to assess 24-h mosquito mortality after exposure to transfluthrin. Mortality was measured for ten of the 32 nights. A third volunteer recorded wind speed using a hand-held anemometer (840003, Sper Scientific) and temperature for each hour just outside the SFS.

### Knock-down test: closed cylinder method

**Figure 4:**
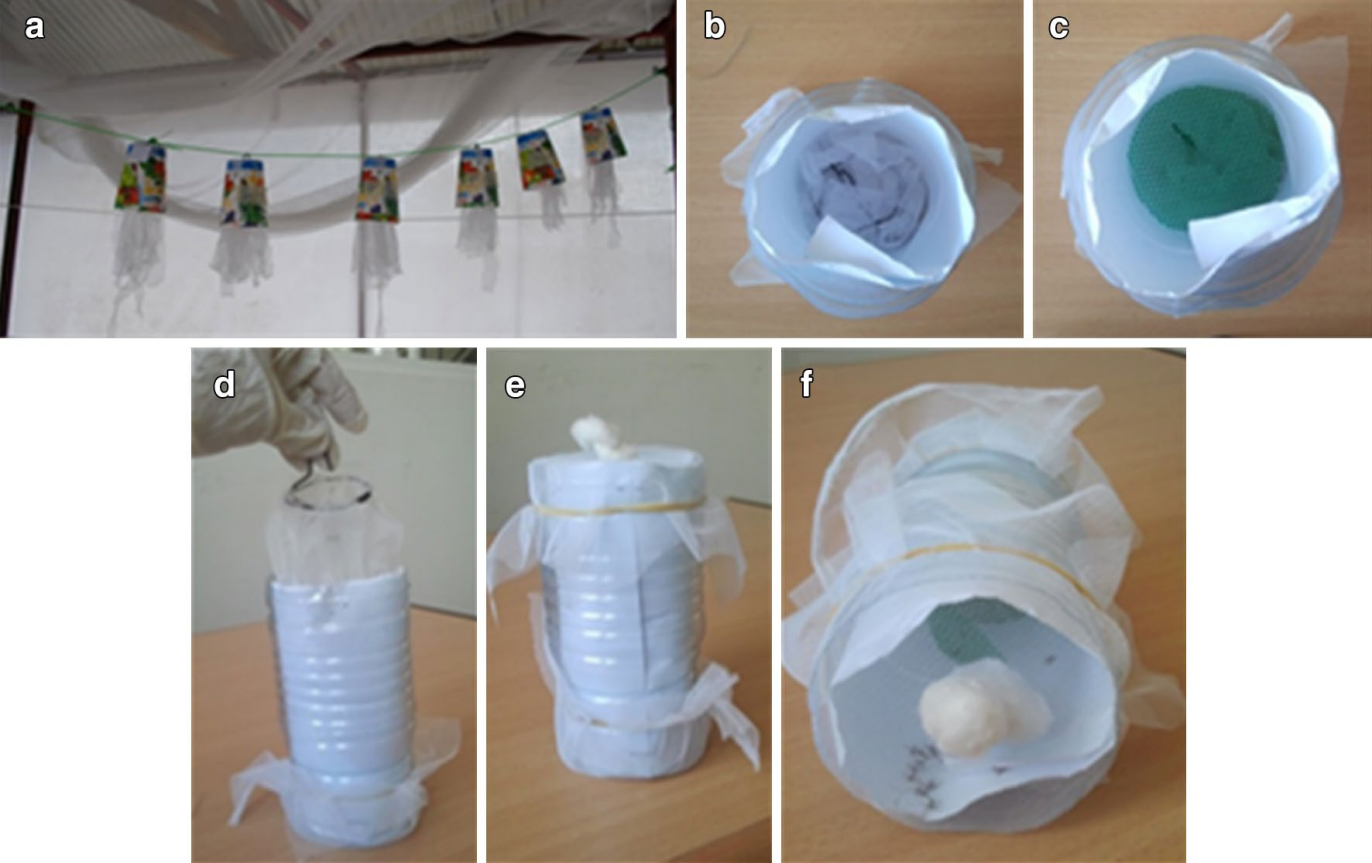
Knock-down test. **a** Polyester strips treated with transfluthrin were kept in half paper cups and hung from a thread to mimic the light and ventilation condition in the modified MLBs. **b**, **d** The polyester strips were placed at the bottom of the plastic bottle, and covered with a plastic mesh to avoid contact between the strips and the mosquitoes (**c**). **e** Both sides of the cylinder were closed using a net. **f** Example of an experiment with polyester strips treated with 90 mg transfluthrin. After a few minutes all mosquitoes were knocked down at the base.

Polyester strips are an efficient means of delivering volatile odours [41]. To test the longevity of transfluthrin on the substrate, a knock-down test was performed with strips that had been stored for varying number of days since impregnation. Polyester strips were collected after each experiment, kept in cut paper cups and hung in similar conditions to those in the MLBs, i.e., a space where wind could freely circulate but where the impregnated strips were partially protected from sun-light (Figure 4a).

A knock-down test was performed with the polyester strips 24 h, 1, 2, and 3 weeks after being impregnated with transfluthrin. The knock-down test protocol followed is a modified version of the cylinder method [42]. A 1.5-l plastic water bottle was cut to a cylinder. A white paper sheet was folded in cylindrical shape and the polyester strips to be tested were placed at the bottom of the bottle (Figure 4b, d). A plastic mesh was applied to the top to ensure no direct contact between the polyester strips and the mosquitoes (Figure 4c). Although there is no contact between the strips and the mosquitoes, the concentration of transfluthrin that they are exposed to within the small, enclosed cylinders is much higher than in the SFS, so a knock-down effect was expected. The top and bottom of the plastic cylinder were closed using a plastic net (Figure 4e). Twenty-five female *An. gambiae s.s.* (Ifakara strain) mosquitoes were introduced into the cylinder and the number of knocked down mosquitoes was counted every minute for 15 min (Figure 4f). The mosquitoes were then kept overnight in paper cups with access to 10% sugar solution to assess 24-h mortality. A new cylinder was used for each test to eliminate the possibility of contamination between tests, and a separate control test was run in parallel to monitor mosquito fitness.

### Protection of participants and ethical approval

The four volunteers performing HLC were experienced male IHI employees ages between 18 and 40 years. They were recruited on written informed consent that explained the risks and benefits of the study, and were free to leave the study without explanation, even though they were employees, as participation was strictly voluntary. The participants were compensated for their time. All participants underwent weekly screening for malaria parasites using SD Bioline PF pan malaria rapid diagnostic test (mRDT) since they were working with mosquitoes in the SFS to ensure no accidental release of an infected mosquito. The study is approved by the Institutional Review Boards of Ifakara Health Institute (IHI/IRB/06-2014), Medical Research Coordinating Committee of the National Institute of Medical Research (NIMR/HQ/R.8a/Vol.IX/1871), and London School of Hygiene and Tropical Medicine (LSHTM7269).

### Statistical analysis

Data were collected on paper forms and entered into an Excel data base and analysed using Stata 11.2 (Stata Corp) with significance level of 0.05 for rejecting the null hypothesis.

For the analysis of the protection and mortality provided by the protective transfluthrin bubble, a multilevel, mixed effect, logistic regression model was used to estimate odds ratios (OR) of different exposures. For estimation of repellent protective efficacy, the response variable was set as the proportion of mosquitoes caught by HLCs out of the total number of mosquitoes released in each compartment (n = 60) and for mortality this was the proportion of dead mosquitoes of those mosquitoes recaptured. For both models, the explanatory variables included the fixed categorical variables ‘treatment’, ‘compartment’ and ‘person’ and their interactions, and the random variable ‘date’ which accounts for random heterogeneity that is caused by fluctuations in temperature and wind speed. Several models were run for each outcome and the final model selected was that with the lowest Akaike’s Information Criterion (AIC). In addition, residuals were plotted using histogram, quantile-quintile plots and comparison with fitted values to ensure appropriateness of model selection.

For the knock-down cylinder test, the proportion of mosquitoes knocked down per minute was modelled using Kaplan–Meier survival curve and the time for knock-down of 50% of the mosquitoes (KD50) calculated. OR of mortality after exposure to knock-down cylinder test was estimated by a multilevel, mixed effect, logistic regression. The response variable was the proportion of mosquitoes dead out of the total number of mosquitoes used in the knock-down test. The explanatory variables were ‘age of strips’ as the fixed categorical variable and ‘date of the initial HLC test’ as random variable.

## Results

### Mosquito landing boxes as efficient dispersers of volatile transfluthrin to create an outdoor repellent protective ‘bubble’

**Figure 5:**
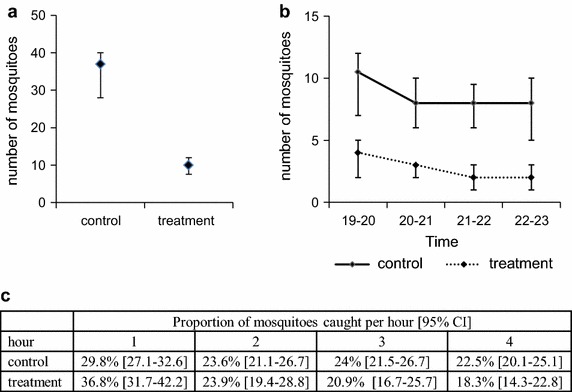
Personal protection provided by the transfluthrin bubble in the SFS. **a** The number of *An. arabiensis* mosquitoes caught under treatment conditions (*right*) was significantly lower compared to the number of mosquitoes caught under control conditions (*left*). Graph showing median and IQR. **b** Distribution of number of mosquitoes caught with human landing catches during each hour of the experiment. Median number of mosquitoes caught per hour and IQR for control and treatment. **c** Proportion of mosquitoes caught per hour.

The protective transfluthrin bubble provided 68.9% protection against *An. arabiensis* mosquito bites. In the repellent arm, 339 out of 1,920 (17.6%) mosquitoes were captured attempting to land on human volunteers, while 1,091 out of 1,920 (56.8%) where caught in the control arm (Table 1; Figure 5a). According to the fitted, mixed effect, logistic regression, the volunteer under the protective transfluthrin bubble had an OR of 0.17 (95% CI 0.09–0.29, p < 0.001) for a mosquito landing compared to the control.

**Table 1:** Effect of 90 mg transfluthrin and sources of experimental bias during the evaluation

	Median IQR mosquito landings/night	OR [95% CI]	z value	p value
Treatment				
Control	37 [28–40]	1	–	–
Treatment	10 [7.5–12.5]	0.17 [0.09–0.29]	−6.20	*<0.001*
Person				
V1	13 [25–36]	1	–	–
V2	12 [8–28.5]	1.04 [0.61–1.79]	0.16	0.873
V3	18.5 [11–28]	0.83 [0.49–1.42]	−0.67	0.504
V4	25.5 [10–38.5]	1.22 [0.71–2.09]	0.72	0.471
Treatment#person				
Treat–V1	13 [9–16.5]	1	–	–
Treat–V2	8 [6.5–10.5]	0.49 [0.21–1.13]	−1.66	0.096
Treat–V3	11 [10–12]	0.88 [0.39–1.98]	−0.30	0.763
Treat–V4	10 [7–12]	0.74 [0.33–1.66]	−0.73	0.467
Compartment				
C1	20.5 [11–37]	1	–	–
C2	15.5 [10–32.5]	1.04 [0.60–1.78]	0.13	0.894
Treatment#compartment				
Treat–C1	11 [7.5–12.5]	1	–	–
Treat–C2	10 [7.5–12.5]	1.00 [0.45–2.22]	0.01	0.994
Person#compartment				
V1–C2	25 [13–37.5]	1	–	–
V2–C2	11.5 [9–17]	0.30 [0.14–0.64]	−3.11	*0.002*
V3–C2	18.5 [11–27.5]	0.56 [0.26–1.21]	−1.47	0.141
V4–C2	23.5 [7–38.5]	1.11 [0.52–2.40]	0.27	0.784
Treatment#person#compartment				
Treat–V1–C2	13 [9–18]	1	–	–
Treat–V2–C2	9.5 [8–11.5]	4.49 [1.40–14.38]	2.53	*0.011*
Treat–V3–C2	11 [10–13.5]	2.08 [0.91–4.75]	1.73	0.083
Treat–V4–C2	7 [6.5–8.5]	0.49 [0.15–1.55]	−1.22	0.223

Statistical parameters estimated by fitting a mixed effect logistic regression model to the data.

Significant *p* values (*p* < 0.05) are in italics.

For both control and treatment, most mosquitoes were caught during the first hour of the experiment between 19.00 and 20.00, although the proportion of mosquitoes was significantly different, being 30% of the mosquitoes in the control, and 37% in the treatment (OR = 1.38, 95% CI 1.07–1.78, p = 0.014). During the following hours there were no significant differences in the proportion of mosquitoes caught oscillating between 18 and 24% in both control and treatment (Figure 5b, c). The best fitting statistical model showed a significant interaction between one of the collectors and compartment 2 when using the control strips as he consistently caught fewer mosquitoes in one of the compartments when he was assigned to control (Table 1) (OR = 0.30, 95% CI 0.14–0.64, p = 0.002) compared to other collectors performing in the same compartment. However, this did not undermine the efficacy of the repellent overall as shown by the final model including all interactions. There was no significant effect of the compartment on the number of mosquitoes caught (OR = 1.04, 95% CI 0.60–1.78, p = 0.894), and neither were there significant differences in the performance of all four volunteers (all p > 0.096; Table 1).

The SFS at the IHI facilities in Bagamoyo was built from east (E) to west (W), following the most common wind direction coming from the sea (E) throughout the year (J Moore, pers. comm). However, the described experiments were performed in the cold season when the wind pattern was shifting and the most common wind direction was from southeast (SE) to northwest (NW). From the total of 32 experimental nights, the wind was blowing from SE to NW for 72 h (56.8%), and at least during 1 h of every experimental day. This wind speed was slightly greater in compartment 1 than in compartment 2 (Figure 3c) although this did not significantly affect mosquito landings. The maximum wind speed was 9.3 km/h and the average speed 0.8 km/h. During the remaining hours of the experiment (43.2%), the wind speed was too low to be measured. The average temperature during the experiment was 24.7°C, with a maximum value of 25.5°C and a minimum of 23.0°C. The average difference in temperature during the 32 experimental days between the start and the end of the experiment was 0.96°C.

### Exposure to volatile transfluthrin causes a low mortality on *An. arabiensis* mosquitoes

For polyester strips impregnated with 90 mg, the pyrethroid insecticide, transfluthrin, the toxic effect was low on *An. arabiensis* mosquitoes. Mosquitoes caught within the transfluthrin bubble showed a significantly greater 24-h mortality of 17% (95% CI 10–26.2%) compared to 7.6% (95% CI 4.8–11.1%) in the control bubble (Table 2) (OR = 2.64, 95% CI 1.28–5.44, p = 0.008).

**Table 2:** Toxicity of 90 mg volatile transfluthrin

	Proportion of mosquitoes	% [95% CI]	OR [95% CI]	z value	p value
Control	23/304	7.6% [4.8–11.1]	1	–	–
Treatment	16/94	17% [10–26.2]	2.64 [1.28–5.44]	2.64	0.008

Proportion of death mosquitoes 24 h after the SFS experiment. OR, z value and p value of treatment compared to control estimated by fitting a mixed effect logistic regression.

### Transfluthrin-treated polyester strips are able to knock-down mosquitoes up to 3 weeks after being impregnated, although this protection diminishes with time

**Figure 6:**
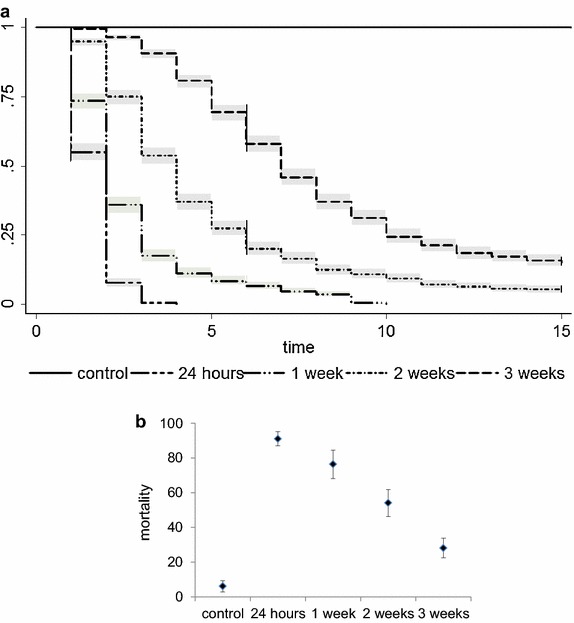
Knock-down and mortality of *Anopheles gambiae s.s.* mosquitoes after exposure to transfluthrin-impregnated strips of different ages using the closed cylinder method. **a** Kaplan–Meier survival estimates of *An. gambiae* *s.s.* knock-down test during 15 min for strips of different age. *Gray areas* show the estimated 95% CI. **b** Assessment of 24-h *An. gambiae s.s.* mortality caused by the polyester strips of different ages after exposure to the knock-down test. Graph showing mean and 95% CI.

Transfluthrin-impregnated strips continued to knock-down mosquitoes up to 3 weeks after being impregnated (Figure 6a), although the time for knock-down of 50% of the mosquitoes (KD50)—as estimated from a Kaplan–Meier survival curve fitted to the data—increased with the age of the strips (Table 3). Twenty-four hours after being impregnated, the KD50 of the strips was 2 min (IQR 1–2 min). The KD50 increased to 7 min (IQR 5–10 min) 3 weeks after the impregnation.

**Table 3:** Effect of age on the estimated time for knock down of 50% of the mosquitoes (KD50) and toxicity of transfluthrin-impregnated strips when mosquitoes are exposed in a cylinder assay

Age of transfluthrin strip	Estimated KD50 (min) [IQR]	24-h mortality [95% CI]	24-h mortality OR [95% CI]	z value	p value
Control	–	6.13% [2.9–9.37]	–	–	–
24 h	2 [1–2]	91% [86.26–95.14]	265.8 [180.41–391.65]	28.23	<0.001
1 week	2 [1–3]	76.29% [68.17–84.41]	72.97 [52.48–101.46]	25.51	<0.001
2 weeks	4 [2–6]	54.04% [46.33–61.75]	24.44 [17.88–33.41]	20.03	<0.001
3 weeks	7 [5–10]	28.15 [22.57–33.72]	6.89 [3.64–7.37]	5.02	<0.001

KD50 in minutes was estimated from Kaplan–Meier survival curve. The 24-h mortality statistical parameters were estimated by fitting a mixed effect logistic regression model.

24-h mortality OR of strips of different age compared to the control strips gets lower the older the strips are. However, 3-week old strips still kill significantly more mosquitoes than control strips.

*OR* odds ratio, *CI* confidence intervals.

Data for 24-h mortality agreed with knock-down test data, showing that 24-h old strips caused 91% mortality (95% CI 87–95) compared to 28% mortality (95% CI 23–34) for 3-week old strips (Figure 6b; Table 3). The OR of mortality after the different exposures compared to the control ranged from 265.8 (95% CI 180.41–391.65) for strips 24-h old to 6.89 (3.64–7.37) for strips 3-weeks old (Table 3).

## Discussion

The effectiveness of MLBs as spatial repellent dispersers to create exposure-free areas where people gather in the evenings was validated in the semi-field, which mimics outdoor environmental conditions. The collected data demonstrate the efficacy of active volatilization to dispense repellents with two MLBs located 5 m either side of a human volunteer, reducing the number of *An. arabiensis* mosquito bites by 68.9%. The effectiveness of active volatilization has been seen with transfluthrin heated by oil lamps [43], mosquito coils [37], with trans-allethrin used in the Thermacell [44] and timed release of essential oils [45]. Active release is important to ensure that repellents are consistently delivered at biologically significant concentrations despite fluctuations in airflow and temperature that affect passive emanation outdoors.

Other studies using passive emanation with four plastic strips each treated with 5% metofluthrin in a shelter without walls, achieved 60% repellency over 15 weeks [46]. In this study, the four impregnated strips were hung in a square formation at a distance of 1–1.5 m from the human. Similarly, another study reports that using 10 mg of transfluthrin passively emanated with Hessian strips prevented more than 90% of human–mosquito mosquito contacts for several months [22]. In this case, the strip was suspended on wooden poles making approximately 1 m^2^ area surrounding the human. Compared to previous studies, the use of active dispersion in this experiment allows an increase in the area protected, even though dose per cubic meter was far lower. Unfortunately, it was not possible to measure the exact airborne concentrations due to their low levels in the air [24]. By increasing the dose or careful formulation of active ingredient used, it is envisaged that the MLB emanation device could be made more efficient and longer lasting, especially as it relies on active dispersion by an automated solar-powered mechanism. Using a photoswitch so that the battery powers the fan at dusk and use of a 4-h battery means that the emanation of the transfluthrin begins when it is needed, i.e., when evening mosquito activity begins, and is switched off when people go to bed. This time-targeted application method means that there is less loss of insecticide during the day and minimal contamination of non-target organisms such as pollinators. The insecticide remains within the odour-dispensing tube where it is not exposed to UV light or air movement, further prolonging the active life of the device. Additionally, MLBs are electricity independent because they are powered by a solar panel that can be used to charge other domestic devices, such as basic lighting or mobile phones. This could positively impact its uptake and maintenance since it provides additional advantages to the target user population.

*Anopheles arabiensis* is one of the most important exophagic vectors in sub-Saharan Africa. It shows flexible behaviour that ranges from endo- to exophily [3, 47, 48], and from highly anthropophilic to zoophagic [49–51]. Because of its capacity to evade control by LLINs and IRS, its relative abundance and importance as malaria vector is increasing in many parts of Africa. Moreover, its biting behaviour can also coincide with the hours when people are carrying out outdoor activities [13, 52]. Previous reports have shown that the most common outdoor activities at this time in sub-Saharan Africa are cooking, eating, watching television, telling stories, and buying and selling foodstuffs [13] that mainly occur close to the house. With the MLB experimental design, it has been possible to create a protective area equivalent to the peri-domestic area commonly used in the rural tropics during 4 h from 19.00 to 23.00, when most of the evening outdoor human activities occur. The study shows how contact between *An. arabiensis* and humans could be greatly reduced in a peri-domestic space during the hours of the vectors’ evening activity, potentially preventing malaria transmission. Night workers in a fixed location, such as market sellers, could also be protected, but other complementary mobile interventions should be implemented if the worker needs to move around, as is the case for many people that have occupational exposure to disease such as forest workers. Further investigation is required to determine appropriate transfluthrin concentrations and MLB distribution depending on the size of the areas and number of people to be targeted. Furthermore, the effect on other evening outdoor-biting malaria vectors that are not completely controlled through LLINs and IRS including *Anopheles darlingi*, *Anopheles dirus*, *Anopheles minimus* and *Anopheles maculatus* should be assessed.

The outcome of this study was a clear decrease in mosquito landings. Previous work on the effect of airborne pyrethroids on different parameters of malaria transmission [37] indicates that transfluthrin prevents human–vector contact indoors by deterring mosquitoes (preventing mosquitoes from entering human dwellings) and inhibiting blood-feeding behaviour. From the experimental design used in this study it is not possible to conclude if the effect of the volatile transfluthrin was to deter mosquitoes from entering the protective bubble or from landing on hosts. There might have been an additional blood feeding inhibition effect [37], but most of the mosquitoes in this study were caught before starting to blood-feed as per HLC guidelines. This study also shows a low toxic effect of volatile transfluthrin inducing 17% mortality (compared to 7.6% mortality in the control). This means that the use of repellent MLBs could affect several entomological parameters that influence malaria transmission. The term vectorial capacity describes this relationship between different entomological parameters [53] and is defined as the expected number of new human malaria infections disseminated per human per day by a mosquito population from a single human case. The equation includes mosquito abundance (m), mosquito daily survival (p) (mosquitoes must live long enough for parasites to develop to the infective stage) and frequency of contacts between mosquito and humans (ma) [54]. Theoretically, all of these parameters would be affected by the repellent MLBs resulting in reduced malaria transmission. The toxic effect of airborne pyrethroids was also reported by Ogoma et al. [37]. In this study, transfluthrin coils caused more than 60% mortality in experimental huts within a SFS, but only 2% in experimental huts in the field. Authors related these differences to the fact that mosquitoes spent more time within huts in the SFS due to the presence of an unprotected human—whereas humans were protected by an untreated bednet in the field experiment and therefore mosquitoes were probably leaving the huts quicker to continue host seeking. This should be taken into account when assessing the real impact of repellent MLBs on malaria transmission because in the field mosquitoes will spend a very short time under the transfluthrin bubble and therefore toxicity might be very low. The fact that in this study, the same strips used within the repellent MLBs induced 90% mortality when mosquitoes were exposed to them in small plastic cylinder for 15 min (Figure 6b) supports the dose-dependent toxicity of transfluthrin and highlights the necessity of field evaluation once the system is optimised to evaluate the real impact of repellent volatile transfluthrin on malaria transmission.

On the other hand, sub-lethal effects of airborne pyrethroids have been previously reported [24, 27, 37]. Even when airborne pyrethroids do not cause toxicity, sub-lethal doses can affect adult longevity, reproductive potential, flying activity, post-exposure blood feeding behaviour, and parasitic charges [55, 56]. There is evidence that olfactory pathways may be affected at low doses, independently of the toxic mechanism caused by the inhibition of sodium channels at higher concentrations [57]. This implies that airborne pyrethroids might still be used even in areas with pyrethroid-resistant mosquito vectors, and this has been recently demonstrated with metofluthrin mosquito coils against highly pyrethroid-resistant *Culex quinquefasciatus* in Benin (N’Guessan, pers. comm). However, a recent study suggests that insensitivity to sub-lethal doses of transfluthrin used as a spatial repellent in the dengue vector *Aedes aegypti* are heritable and correlate to reduced susceptibility to toxic doses of transfluthrin in CDC bottle assays [58]. Furthermore, recent research from Tanzania (Moore, pers. com) indicates that highly multiple-resistant (permethrin, deltamethrin, lambda cyhalothrin, DDT, and bendiocarb) *An. funestus* continue to land on HLC volunteers in the presence of transfluthrin, whereas less resistant (susceptible to bendiocarb and DDT but resistant to pyrethroids) *An. arabiensis* and *Mansonia uniformis*/*africana* do not land on the same HLC volunteers in the same houses. Therefore, the real impact of MLB dispersed volatile transfluthrin is likely to be highly dependent on mosquito population life history characteristics and mechanisms of insecticide resistance.

Importantly, before considering implementation of outdoor spatial repellents as vector control interventions, it is essential to avoid mosquitoes that are repelled from protected areas being able to divert to biting unprotected people [59]. One potential strategy is to combine spatial repellents with MLBs used for lure and kill [13]. Odour-baited MLBs were able to attract large numbers of *An. arabiensis* and when used with a contact toxicant pirimiphos methyl at 5%, up to 50% of the mosquitoes visiting the MLBs were killed. Therefore, a solution to prevent the diversion of repelled mosquitoes to unprotected areas would be to put odour-baited luring and killing MLBs in the vicinity of the protective bubble created by the repellent MLBs. This would attract and kill diverted mosquitoes and stop them from biting unprotected people. However, the lure and kill MLBs must not be located too close to the repellent MLBs or the peridomestic area, in case mosquitoes are lured towards places where people are congregated, which could lower the protective efficacy of the repellent MLBs or even increase human exposure to mosquitoes. Using this approach, if correctly applied, even if repellent MLBs coverage is not complete in a village, it should not mean an additional risk for the unprotected inhabitants.

The importance of understanding how environmental factors affect outdoor interventions is an essential consideration of this study. The interaction of compartment, person and treatment significantly influenced mosquito landings (Table 1), indicating that the effect of the transfluthrin protective bubble varies depending on the compartment and on the person within this compartment. The variability in mosquito attractiveness of different humans has been extensively reported [60–63] and might modulate the protective effect of the transfluthrin bubble. It is for this reason that it is important to evaluate repellents on a number of people, to ensure that the repellent is effective even for those who are normally highly attractive to mosquitoes in the absence of repellent. Another source of variation commonly encountered when evaluating repellents is location, because air flow and sources of competing kairomones are heterogenous in space. This was demonstrated in the final model as an additional confounder, and underlines the importance of controlling for such effects in repellents evaluations through rotations and balanced design. Despite all the sources of variation, the transfluthrin bubble still provides protection when all interactions are included (Table 1, p < 0.001). Another important consideration of this study is the length of time the same transfluthrin-impregnated strips can be used without being replaced. The idea behind MLBs is that they are simple and effective devices to be used in middle- and low-income countries by local people. The longer the strips retain their repellency, the easier it is for the users to adhere to the intervention since they do not need to replace them as often. To test the effectiveness of the strips, a knock-down test of the strips was performed 24 h, 1, 2, and 3 weeks after being used for the experiment. Although the mode of action of transfluthrin to knockdown mosquitoes is different to the repellent molecular pathway, it is well understood that knock down time and repellency correlate in tests of pyrethroid mosquito coils [42]. The purpose of these tests was to evaluate how the effectiveness of the strips declines with time as a result of gradual loss of transfluthrin. Impregnated strips were kept in conditions as similar as possible to the odour compartment of MLBs to simulate the conditions on the field. These results showed that the knock-down capacity of the strips was high up to 2 weeks after being impregnated, after which it decreased slightly. Future work should focus on maximizing longevity of spatial repellent strips to maximize outdoor protection at lowest cost.

## Conclusion

This study describes a potential means of deploying repellents to provide high levels of personal protection against outdoor-biting mosquitoes without the need for daily user compliance. The dispersal of transfluthrin using two MLBs separated 5 m from a human reduced mosquito-biting rate by 68.9% under outdoor conditions. The materials used to construct MLBs are economical and easily available, and their functioning is user-friendly and electricity independent. These features offered by MLBs are important considerations for the success of outdoor spatial repellency as a promising additional tool to tackle residual malaria transmission in middle- and low-income countries.

## References

[1] WHO (2014). World Malaria Report 2014.

[2] Durnez L, Coosemans M (2013) Residual transmission of malaria: an old issue for new approaches. In: Manguin S (ed) Anopheles mosquitoes—new insights into malaria vectors, chap 21. InTech, pp 671–704

[3] Fontenille D, Simard F (2004). Unravelling complexities in human malaria transmission dynamics in Africa through a comprehensive knowledge of vector populations. Comp Immunol Microbiol Infect Dis.

[4] Durnez L, Mao S, Denis L, Roelants P, Sochantha T, Coosemans M (2013). Outdoor malaria transmission in forested villages of Cambodia. Malar J.

[5] Bayoh MN, Mathias DK, Odiere MR, Mutuku FM, Kamau L, Gimnig JE (2010). *Anopheles gambiae*: historical population decline associated with regional distribution of insecticide-treated bed nets in western Nyanza Province, Kenya. Malar J.

[6] Lindblade KA, Gimnig JE, Kamau L, Hawley WA, Odhiambo F, Olang G (2006). Impact of sustained use of insecticide-treated bednets on malaria vector species distribution and culicine mosquitoes. J Med Entomol.

[7] Mutuku FM, King CH, Mungai P, Mbogo C, Mwangangi J, Muchiri EM (2011). Impact of insecticide-treated bed nets on malaria transmission indices on the south coast of Kenya. Malar J.

[8] Parker BS, Paredes Olortegui M, Penataro Yori P, Escobedo K, Florin D, Rengifo Pinedo S (2013). Hyperendemic malaria transmission in areas of occupation-related travel in the Peruvian Amazon. Malar J.

[9] Sinka ME, Rubio-Palis Y, Manguin S, Patil AP, Temperley WH, Gething PW (2010). The dominant Anopheles vectors of human malaria in the Americas: occurrence data, distribution maps and bionomic precis. Parasite Vectors.

[10] Sinka ME, Bangs MJ, Manguin S, Chareonviriyaphap T, Patil AP, Temperley WH (2011). The dominant Anopheles vectors of human malaria in the Asia-Pacific region: occurrence data, distribution maps and bionomic precis. Parasite Vectors.

[11] Alonso PL, Besansky NJ, Burkot TR, Collins FH, Hemingway J, James AA (2011). A research agenda for malaria eradication: vector control. PLoS Med.

[12] Okumu FO, Killeen GF, Ogoma S, Biswaro L, Smallegange RC, Mbeyela E (2010). Development and field evaluation of a synthetic mosquito lure that is more attractive than humans. PLoS One.

[13] Matowo NS, Moore J, Mapua S, Madumla EP, Moshi IR, Kaindoa EW (2013). Using a new odour-baited device to explore options for luring and killing outdoor-biting malaria vectors: a report on design and field evaluation of the Mosquito Landing Box. Parasite Vectors.

[14] Rowland M, Durrani N, Kenward M, Mohammed N, Urahman H, Hewitt S (2001). Control of malaria in Pakistan by applying deltamethrin insecticide to cattle: a community-randomised trial. Lancet.

[15] Chaki PP, Govella NJ, Shoo B, Hemed A, Tanner M, Fillinger U (2009). Achieving high coverage of larval-stage mosquito surveillance: challenges for a community-based mosquito control programme in urban Dar es Salaam, Tanzania. Malar J.

[16] Fillinger U, Kannady K, William G, Vanek MJ, Dongus S, Nyika D (2008). A tool box for operational mosquito larval control: preliminary results and early lessons from the urban malaria control programme in Dar es Salaam, Tanzania. Malar J.

[17] Fillinger U, Ndenga B, Githeko A, Lindsay SW (2009). Integrated malaria vector control with microbial larvicides and insecticide-treated nets in western Kenya: a controlled trial. Bull World Health Organ.

[18] Tusting LS, Thwing J, Sinclair D, Fillinger U, Gimnig J, Bonner KE (2013). Mosquito larval source management for controlling malaria. Cochrane Database Syst Rev.

[19] Castro MC, Tsuruta A, Kanamori S, Kannady K, Mkude S (2009). Community-based environmental management for malaria control: evidence from a small-scale intervention in Dar es Salaam, Tanzania. Malar J.

[20] Castro MC, Kanamori S, Kannady K, Mkude S, Killeen GF, Fillinger U (2010). The importance of drains for the larval development of lymphatic filariasis and malaria vectors in Dar es Salaam, United Republic of Tanzania. PLoS Negl Trop Dis.

[21] Deressa W, Yihdego YY, Kebede Z, Batisso E, Tekalegne A, Dagne GA (2014). Effect of combining mosquito repellent and insecticide treated net on malaria prevalence in Southern Ethiopia: a cluster-randomised trial. Parasite Vectors.

[22] Ogoma SB, Ngonyani H, Simfukwe ET, Mseka A, Moore J, Killeen GF (2012). Spatial repellency of transfluthrin-treated hessian strips against laboratory-reared *Anopheles arabiensis* mosquitoes in a semi-field tunnel cage. Parasite Vectors.

[23] Zhang L, Jiang Z, Tong J, Wang Z, Han Z, Zhang J (2010). Using charcoal as base material reduces mosquito coil emissions of toxins. Indoor Air.

[24] Achee N, Masuoka P, Smith P, Martin N, Chareonviryiphap T, Polsomboon S (2012). Identifying the effective concentration for spatial repellency of the dengue vector *Aedes aegypti*. Parasite Vectors.

[25] Grieco JP, Achee NL, Chareonviriyaphap T, Suwonkerd W, Chauhan K, Sardelis MR (2007). A new classification system for the actions of IRS chemicals traditionally used for malaria control. PLoS One.

[26] Ogoma SB, Moore SJ, Maia MF (2012). A systematic review of mosquito coils and passive emanators: defining recommendations for spatial repellency testing methodologies. Parasite Vectors.

[27] Hill N, Zhou HN, Wang P, Guo X, Carneiro I, Moore SJ (2014). A household randomized, controlled trial of the efficacy of 0.03% transfluthrin coils alone and in combination with long-lasting insecticidal nets on the incidence of *Plasmodium falciparum* and *Plasmodium vivax* malaria in Western Yunnan Province, China. Malar J.

[28] Syafruddin D, Bangs MJ, Sidik D, Elyazar I, Asih PB, Chan K (2014). Impact of a spatial repellent on malaria incidence in two villages in Sumba, Indonesia. Am J Trop Med Hyg.

[29] Sangoro O, Kelly AH, Mtali S, Moore SJ (2014). Feasibility of repellent use in a context of increasing outdoor transmission: a qualitative study in rural Tanzania. Malar J.

[30] Maia MF, Moore SJ (2011). Plant-based insect repellents: a review of their efficacy, development and testing. Malar J.

[31] Goodyer LI, Croft AM, Frances SP, Hill N, Moore SJ, Onyango SP (2010). Expert review of the evidence base for arthropod bite avoidance. J Travel Med.

[32] Dadzie S, Boakye D, Asoala V, Koram K, Kiszewski A, Appawu M (2013). A community-wide study of malaria reduction: evaluating efficacy and user-acceptance of a low-cost repellent in northern Ghana. Am J Trop Med Hyg.

[33] Kroeger A, Gerhardus A, Kruger G, Mancheno M, Pesse K (1997). The contribution of repellent soap to malaria control. Am J Trop Med Hyg.

[34] Hill N, Lenglet A, Arnéz AM, Carneiro I (1023). Plant based insect repellent and insecticide treated bed nets to protect against malaria in areas of early evening biting vectors: double blind randomised placebo controlled clinical trial in the Bolivian Amazon. BMJ.

[35] Rowland M, Downey G, Rab A, Freeman T, Mohammad N, Rehman H (2004). DEET mosquito repellent provides personal protection against malaria: a household randomized trial in an Afghan refugee camp in Pakistan. Trop Med Int Health.

[36] Wilson AL, Chen-Hussey V, Logan JG, Lindsay SW (2014). Are topical insect repellents effective against malaria in endemic populations? A systematic review and meta-analysis. Malar J.

[37] Ogoma SB, Lorenz LM, Ngonyani H, Sangusangu R, Kitumbukile M, Kilalangongono M (2014). An experimental hut study to quantify the effect of DDT and airborne pyrethroids on entomological parameters of malaria transmission. Malar J.

[38] Ferguson HM, Ng’habi KR, Walder T, Kadungula D, Moore SJ, Lyimo I (2008). Establishment of a large semi-field system for experimental study of African malaria vector ecology and control in Tanzania. Malar J.

[39] Gimnig JE, Walker ED, Otieno P, Kosgei J, Olang G, Ombok M (2013). Incidence of malaria among mosquito collectors conducting human landing catches in western Kenya. Am J Trop Med Hyg.

[40] WHO (1975). Manual on practical entomology in malaria, part II.

[41] Mweresa CK, Mukabana WR, Omusula P, Otieno B, Gheysens T, Takken W (2014). Evaluation of textile substrates for dispensing synthetic attractants for malaria mosquitoes. Parasite Vectors.

[42] Chadwick PR (1975). The activity of some pyrethroids, DDT and lindane in smoke from coils for biting inhibition, knockdown and kill of mosquitoes (Diptera, Culicidae). Bull Entomol Res.

[43] Pates HV, Line JD, Keto AJ, Miller JE (2002). Personal protection against mosquitoes in Dar es Salaam, Tanzania, by using a kerosene oil lamp to vaporize transfluthrin. Med Vet Entomol.

[44] Alten B, Caglar SS, Simsek FM, Kaynas S, Perich MJ (2003). Field evaluation of an area repellent system (Thermacell) against *Phlebotomus papatasi* (Diptera: Psychodidae) and *Ochlerotatus caspius* (Diptera: Culicidae) in Sanliurfa Province, Turkey. J Med Entomol.

[45] Revay EE, Junnila A, Kline DL, Xue RD, Bernier UR, Kravchenko VD (2012). Reduction of mosquito biting pressure by timed-release 0.3% aerosolized geraniol. Acta Trop.

[46] Kawada H, Maekawa Y, Takagi M (2005). Field trial of the spatial repellency of metofluthrin-impregnated plastic strip against mosquitoes in shelters without walls (Beruga) in Lombok, Indonesia. J Vector Ecol.

[47] Fornadel CM, Norris LC, Glass GE, Norris DE (2010). Analysis of *Anopheles arabiensis* blood feeding behavior in southern Zambia during the two years after introduction of insecticide-treated bed nets. Am J Trop Med Hyg.

[48] Okello PE, Bortel WV, Byaruhanga AM, Correwyn A, Roelants P, Talisuna A (2006). Variation in malaria transmission intensity in seven sites throughout Uganda. Am J Trop Med Hyg.

[49] Kent RJ, Thuma PE, Mharakurwa S, Norris DE (2007). Seasonality, blood feeding behavior, and transmission of *Plasmodium falciparum* by *Anopheles arabiensis* after an extended drought in southern Zambia. Am J Trop Med Hyg.

[50] Mwangangi JM, Mbogo CM, Nzovu JG, Githure JI, Yan G, Beier JC (2003). Blood-meal analysis for anopheline mosquitoes sampled along the Kenyan coast. J Am Mosq Control Assoc.

[51] Mnzava AE, Mutinga MJ, Staak C (1994). Host blood meals and chromosomal inversion polymorphism in *Anopheles arabiensis* in the Baringo District of Kenya. J Am Mosq Control Assoc.

[52] Geissbühler Y, Chaki P, Emidi B, Govella NJ, Shirima R, Mayagaya V (2007). Interdependence of domestic malaria prevention measures and mosquito–human interactions in urban Dar es Salaam, Tanzania. Malar J.

[53] Garrett-Jones C (1964). The human blood index of malaria vectors in relation to epidemiological assessment. Bull World Health Organ.

[54] MacDonald G (1956). Epidemiological basis of malaria control. Bull World Health Organ.

[55] Hill N (2003). Effects of sublethal doses of pyrethroids on malaria vectors.

[56] Cohnstaedt LW, Allan SA (2011). Effects of sublethal pyrethroid exposure on the host-seeking behavior of female mosquitoes. J Vector Ecol.

[57] Bohbot JD, Fu L, Le TC, Chauhan KR, Cantrell CL, Dickens JC (2011). Multiple activities of insect repellents on odorant receptors in mosquitoes. Med Vet Entomol.

[58] Wagman JM, Achee NL, Grieco JP (2015). Insensitivity to the spatial repellent action of transfluthrin in *Aedes aegypti*: a heritable trait associated with decreased insecticide susceptibility. PLoS Negl Trop Dis.

[59] Maia MF, Abonuusum A, Lorenz LM, Clausen P-H, Bauer B, Garms R (2012). The effect of deltamethrin-treated net fencing around cattle enclosures on outdoor-biting mosquitoes in Kumasi, Ghana. PLoS One.

[60] Mukabana WR, Takken W, Coe R, Knols BGJ (2002). Host-specific cues cause differential attractiveness of Kenyan men to the African malaria vector *Anopheles gambiae*. Malar J.

[61] Qiu YT, Smallegange RC, Loon JJAV, Braak CJFT, Takken W (2006). Interindividual variation in the attractiveness of human odours to the malaria mosquito *Anopheles gambiae s. s*. Med Vet Entomol.

[62] Qiu YT, Smallegange RC, Loon JJAV, Takken W (2011). Behavioural responses of *Anopheles gambiae* sensu stricto to components of human breath, sweat and urine depend on mixture composition and concentration. Med Vet Entomol.

[63] Smallegange RC, Verhulst NO, Takken W (2011). Sweaty skin: an invitation to bite?. Trends Parasitol.

